# 5-((Meth­oxy­imino)­{2-[(2-methyl­phen­oxy)meth­yl]phen­yl}meth­yl)-*N*-phenyl-1,3,4-oxa­diazol-2-amine

**DOI:** 10.1107/S1600536814003821

**Published:** 2014-02-26

**Authors:** Devinder K. Sharma, Chetan S. Shripanavar, Sumati Anthal, Vivek K. Gupta, Rajni Kant

**Affiliations:** a Post-Graduate Department of Physics & Electronics, University of Jammu, Jammu Tawi 180 006, India; b National Research Centre for Grapes, Pune 412 307, India

## Abstract

In the title mol­ecule, C_24_H_22_N_4_O_3_, the plane of the oxa­diazole ring forms a dihedral angle of 32.41 (12)° with that of the phenyl ring and dihedral angles of 74.51 (10) and 56.38 (10)° with the planes of the benzene rings. In the crystal, pairs of N—H⋯N hydrogen bonds link molecules into inversion dimers featuring *R*_2_^2^(8) graph-set motifs.

## Related literature   

For background information and applications of oxa­diazole derivatives, see: Schnurch *et al.* (2006 ▶); Crabtree (2005 ▶); Venkatakrishnan *et al.* (2000 ▶); Brown *et al.* (1992 ▶). For biological activity of oxa­diazole derivatives, see: Omar *et al.* (1996 ▶); Talawar *et al.* (1996 ▶); Hamad *et al.* (1996 ▶); Tully *et al.* (1991 ▶); Barry *et al.* (1991 ▶); Ladduwahetty *et al.* (1996 ▶); Borg *et al.* (1999 ▶). For standard bond lengths, see: Allen *et al.* (1987 ▶). For a related structure, see: Shang *et al.* (2005 ▶).
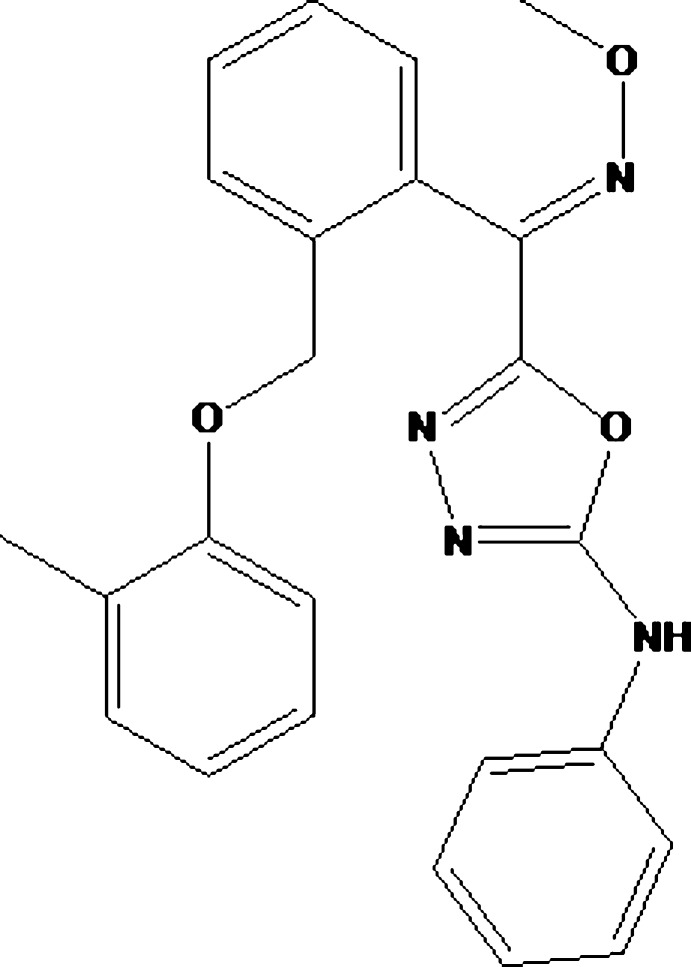


## Experimental   

### 

#### Crystal data  

C_24_H_22_N_4_O_3_
*M* *_r_* = 414.46
Triclinic, 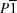

*a* = 7.0629 (4) Å
*b* = 12.5553 (9) Å
*c* = 13.3705 (11) Å
α = 68.321 (7)°
β = 83.678 (6)°
γ = 78.567 (6)°
*V* = 1079.04 (13) Å^3^
*Z* = 2
Mo *K*α radiation
μ = 0.09 mm^−1^
*T* = 293 K
0.30 × 0.20 × 0.20 mm

#### Data collection  

Oxford Diffraction Xcalibur Sapphire3 diffractometer
Absorption correction: multi-scan (*CrysAlis RED*; Oxford Diffraction, 2010 ▶) *T*_min_ = 0.874, *T*_max_ = 1.000
7584 measured reflections
4233 independent reflections
2679 reflections with *I* > 2σ(*I*)
*R*_int_ = 0.023

#### Refinement  

*R*[*F* ^2^ > 2σ(*F* ^2^)] = 0.047
*wR*(*F* ^2^) = 0.121
*S* = 1.01
4233 reflections
286 parameters
H atoms treated by a mixture of independent and constrained refinement
Δρ_max_ = 0.19 e Å^−3^
Δρ_min_ = −0.15 e Å^−3^

### 

Data collection: *CrysAlis PRO* (Oxford Diffraction, 2010 ▶); cell refinement: *CrysAlis PRO*; data reduction: *CrysAlis PRO*; program(s) used to solve structure: *SHELXS97* (Sheldrick, 2008 ▶); program(s) used to refine structure: *SHELXL97* (Sheldrick, 2008 ▶); molecular graphics: *ORTEP-3 for Windows* (Farrugia, 2012 ▶) and *PLATON* (Spek, 2009 ▶); software used to prepare material for publication: *PLATON*.

## Supplementary Material

Crystal structure: contains datablock(s) I, New_Global_Publ_Block. DOI: 10.1107/S1600536814003821/lh5692sup1.cif

Structure factors: contains datablock(s) I. DOI: 10.1107/S1600536814003821/lh5692Isup2.hkl

Supporting information file. DOI: 10.1107/S1600536814003821/lh5692Isup3.cml

CCDC reference:

Additional supporting information:  crystallographic information; 3D view; checkCIF report

## Figures and Tables

**Table 1:** Hydrogen-bond geometry (Å, °)

*D*—H⋯*A*	*D*—H	H⋯*A*	*D*⋯*A*	*D*—H⋯*A*
N6—H6′⋯N3^i^	0.93 (2)	1.99 (2)	2.922 (2)	174

Symmetry code: (i) 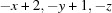.

**Fig. 1.**
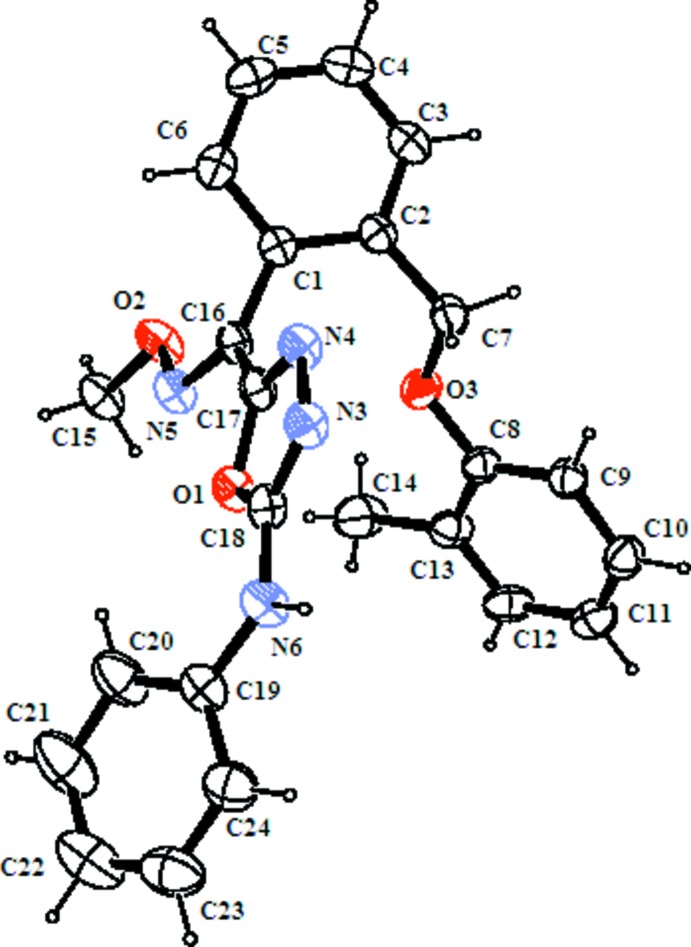
The molecular structure of the title compound with ellipsoids drawn at the 40% probability level. H atoms are shown as small spheres of arbitrary radii.

**Fig. 2.**
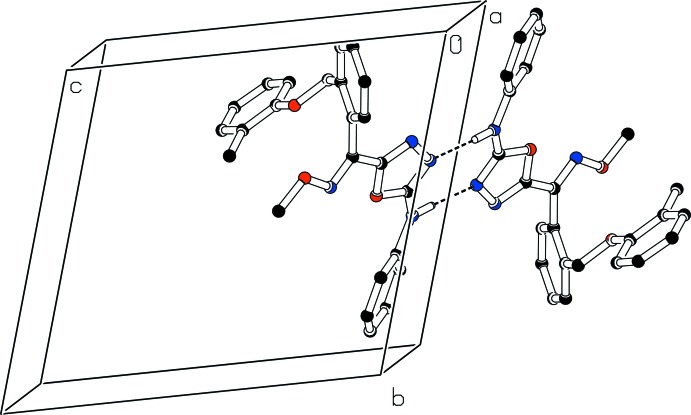
Part of the crystal structure with hydrogen bonds shown as dashed lines showing and inversion dimer.

## supplementary crystallographic information

### 1. Comment

Derivatives of oxadiazole systems are a growing research interest, as they are precursors to functional N-heterocyclic compounds, as well as being used in pharmaceuticals as metabolically stable surrogates and photographically active systems (Schnurch *et al.*, 2006; Crabtree, 2005; Venkatakrishnan, *et al.*, 2000). Symmetrical and unsymmetrical 1,3,4-oxadiazoles have been reported to be versatile compounds displaying a variety of biological effects, which include anti-inflammatory (Omar *et al.*, 1996), antifungal (Talawar *et al.*, 1996) and antimicrobial (Hamad *et al.*, 1996) activities. They have been utilized as bioisosteres of the carboxamide moiety in benzodiazepine receptor agonists, muscarinic receptor agonists, NK1 receptor antagonists, and Phe–Gly peptidomimetics (Tully *et al.*, 1991; Barry *et al.*, 1991; Ladduwahetty *et al.*, 1996; Borg *et al.*, 1999). Moreover, oxadiazole derivatives have been widely used as electron-conducting and hole-blocking materials in moleculebased as well as polymeric light-emitting devices (LEDs) due to the electron-deficient and favourable electron-transport properties of the oxadiazole rings (Brown *et al.*, 1992).

In the molecule of the title compound, Fig.1, bond lengths are in normal ranges (Allen *et al.*, 1987) and are comparable with a related structure (Shang *et al.*, 2005). The oxadiazole ring (O1/N3/N4/C17/C18) forms a dihedral angle of 32.41 (12)° with the phenyl ring (C19-C24) and dihedral angles 74.51 (10) and 56.38 (10)° with the benzene rings (C1-C6 and C8-C13, respectfully). In the crystal, pairs of N—H···N hydrogen bonds form inversion dimers (Fig. 2).

### 2. Experimental

To a suspension of 2-[(2-(methoxyimino)-2-{2-[(2-methylphenoxy)methyl]phenyl}acetyl]-*N* -phenylhydrazinecarbothioamide (2.240 g, 5 mmol) in ethanol, potassium hydroxide solution (4 N, 30 ml) was added with cooling and shaking. A solution of 10% iodine in potassium iodide was added drop wise with stirring till the color of iodine persisted. The mixture was refluxed on a water bath for 4 h, and then left to cool. The separated solid was filtered off washed with water, by the process of slow evaporation recrystallized it from methanol.

### 3. Refinement

H6' attached to N6 was located in a difference Fourier map and refined isotropically. The remaining H atoms were positioned geometrically and were treated as riding on their parent C atoms, with C—H distances of 0.93–0.97 Å; and with *U*_iso_(H) = 1.2*U*_eq_(C), except for the methyl groups where *U*_iso_(H) = 1.5*U*_eq_(C),.

### Crystal data

**Table:**

C_24_H_22_N_4_O_3_	*Z* = 2
*M**_r_* = 414.46	*F*(000) = 436
Triclinic, *P*1	*D*_x_ = 1.276 Mg m^−^^3^
Hall symbol: -P 1	Mo *K*α radiation, λ = 0.71073 Å
*a* = 7.0629 (4) Å	Cell parameters from 2043 reflections
*b* = 12.5553 (9) Å	θ = 3.9–25.8°
*c* = 13.3705 (11) Å	µ = 0.09 mm^−^^1^
α = 68.321 (7)°	*T* = 293 K
β = 83.678 (6)°	Block, colourless
γ = 78.567 (6)°	0.30 × 0.20 × 0.20 mm
*V* = 1079.04 (13) Å^3^	

### Data collection

**Table:**

Oxford Diffraction Xcalibur Sapphire3 diffractometer	4233 independent reflections
Radiation source: fine-focus sealed tube	2679 reflections with *I* > 2σ(*I*)
Graphite monochromator	*R*_int_ = 0.023
Detector resolution: 16.1049 pixels mm^-1^	θ_max_ = 26.0°, θ_min_ = 3.5°
ω scans	*h* = −8→8
Absorption correction: multi-scan (*CrysAlis RED*; Oxford Diffraction, 2010)	*k* = −15→13
*T*_min_ = 0.874, *T*_max_ = 1.000	*l* = −16→13
7584 measured reflections	

### Refinement

**Table:**

Refinement on *F*^2^	Primary atom site location: structure-invariant direct methods
Least-squares matrix: full	Secondary atom site location: difference Fourier map
*R*[*F*^2^ > 2σ(*F*^2^)] = 0.047	Hydrogen site location: inferred from neighbouring sites
*wR*(*F*^2^) = 0.121	H atoms treated by a mixture of independent and constrained refinement
*S* = 1.01	*w* = 1/[σ^2^(*F*_o_^2^) + (0.0476*P*)^2^] where *P* = (*F*_o_^2^ + 2*F*_c_^2^)/3
4233 reflections	(Δ/σ)_max_ = 0.001
286 parameters	Δρ_max_ = 0.19 e Å^−^^3^
0 restraints	Δρ_min_ = −0.15 e Å^−^^3^

### Special details

**Table:**

Experimental. *CrysAlis PRO*, Oxford Diffraction Ltd., Version 1.171.34.40 (release 27–08-2010 CrysAlis171. NET) (compiled Aug 27 2010,11:50:40) Empirical absorption correction using spherical harmonics, implemented in SCALE3 ABSPACK scaling algorithm.
Geometry. All e.s.d.'s (except the e.s.d. in the dihedral angle between two l.s. planes) are estimated using the full covariance matrix. The cell e.s.d.'s are taken into account individually in the estimation of e.s.d.'s in distances, angles and torsion angles; correlations between e.s.d.'s in cell parameters are only used when they are defined by crystal symmetry. An approximate (isotropic) treatment of cell e.s.d.'s is used for estimating e.s.d.'s involving l.s. planes.
Refinement. Refinement of *F*^2^ against ALL reflections. The weighted *R*-factor *wR* and goodness of fit *S* are based on *F*^2^, conventional *R*-factors *R* are based on *F*, with *F* set to zero for negative *F*^2^. The threshold expression of *F*^2^ > σ(*F*^2^) is used only for calculating *R*-factors(gt) *etc*. and is not relevant to the choice of reflections for refinement. *R*-factors based on *F*^2^ are statistically about twice as large as those based on *F*, and *R*- factors based on ALL data will be even larger.

### Fractional atomic coordinates and isotropic or equivalent isotropic displacement parameters (Å^2^)

**Table:**

	*x*	*y*	*z*	*U*_iso_*/*U*_eq_	
O1	0.59108 (16)	0.51331 (10)	0.15728 (10)	0.0477 (3)	
O2	0.09810 (18)	0.40040 (11)	0.31402 (11)	0.0599 (4)	
N5	0.2555 (2)	0.44773 (13)	0.25488 (13)	0.0503 (4)	
O3	0.57360 (17)	0.21849 (12)	0.42203 (11)	0.0591 (4)	
N3	0.7893 (2)	0.43994 (14)	0.04992 (13)	0.0505 (4)	
C1	0.3357 (2)	0.25405 (15)	0.23721 (14)	0.0408 (4)	
C2	0.4559 (3)	0.15355 (15)	0.29884 (15)	0.0443 (5)	
C17	0.5374 (2)	0.41397 (15)	0.15669 (15)	0.0428 (4)	
N4	0.6492 (2)	0.36807 (13)	0.09560 (13)	0.0503 (4)	
C7	0.6318 (3)	0.15775 (16)	0.34995 (16)	0.0513 (5)	
H7A	0.6962	0.0794	0.3887	0.062*	
H7B	0.7216	0.1971	0.2949	0.062*	
N6	0.8571 (2)	0.60402 (15)	0.07479 (14)	0.0549 (5)	
C16	0.3681 (2)	0.37311 (15)	0.22091 (14)	0.0420 (4)	
C18	0.7501 (3)	0.52194 (16)	0.09016 (15)	0.0448 (5)	
C8	0.7125 (3)	0.22372 (16)	0.48381 (16)	0.0460 (5)	
C9	0.9052 (3)	0.17465 (16)	0.47816 (16)	0.0514 (5)	
H9	0.9485	0.1368	0.4297	0.062*	
C10	1.0330 (3)	0.18281 (17)	0.54587 (18)	0.0599 (6)	
H10	1.1631	0.1512	0.5419	0.072*	
C15	−0.0188 (3)	0.48144 (17)	0.35618 (17)	0.0598 (5)	
H15A	0.0509	0.4915	0.4089	0.090*	
H15B	−0.1353	0.4527	0.3893	0.090*	
H15C	−0.0510	0.5548	0.2988	0.090*	
C3	0.4104 (3)	0.04676 (16)	0.31252 (17)	0.0576 (6)	
H3	0.4885	−0.0206	0.3542	0.069*	
C6	0.1759 (3)	0.24319 (18)	0.19236 (16)	0.0525 (5)	
H6	0.0941	0.3098	0.1525	0.063*	
C13	0.6458 (3)	0.28167 (17)	0.55509 (17)	0.0540 (5)	
C11	0.9692 (3)	0.23681 (19)	0.61822 (18)	0.0621 (6)	
H11	1.0549	0.2403	0.6646	0.074*	
C12	0.7771 (3)	0.28617 (18)	0.62222 (17)	0.0607 (6)	
H12	0.7349	0.3234	0.6713	0.073*	
C19	0.8052 (3)	0.71032 (17)	0.09168 (16)	0.0536 (5)	
C5	0.1362 (3)	0.1356 (2)	0.20575 (18)	0.0628 (6)	
H5	0.0302	0.1298	0.1737	0.075*	
C4	0.2533 (3)	0.0375 (2)	0.26631 (19)	0.0650 (6)	
H4	0.2266	−0.0354	0.2762	0.078*	
C24	0.9531 (4)	0.7683 (2)	0.08959 (18)	0.0709 (6)	
H24	1.0806	0.7354	0.0798	0.085*	
C22	0.7283 (6)	0.9234 (2)	0.1171 (2)	0.1028 (10)	
H22	0.7021	0.9951	0.1258	0.123*	
C23	0.9130 (5)	0.8743 (2)	0.1019 (2)	0.0896 (8)	
H23	1.0137	0.9130	0.0997	0.108*	
C20	0.6179 (3)	0.7598 (2)	0.1058 (2)	0.0837 (8)	
H20	0.5165	0.7223	0.1062	0.100*	
C14	0.4381 (3)	0.3382 (2)	0.5583 (2)	0.0883 (8)	
H14A	0.4059	0.3956	0.4888	0.133*	
H14B	0.4189	0.3748	0.6111	0.133*	
H14C	0.3567	0.2802	0.5771	0.133*	
C21	0.5811 (5)	0.8661 (2)	0.1194 (3)	0.1116 (10)	
H21	0.4543	0.8992	0.1304	0.134*	
H6'	0.975 (3)	0.5895 (19)	0.039 (2)	0.088 (8)*	

### Atomic displacement parameters (Å^2^)

**Table:**

	*U*^11^	*U*^22^	*U*^33^	*U*^12^	*U*^13^	*U*^23^
O1	0.0448 (7)	0.0463 (7)	0.0474 (8)	−0.0087 (6)	0.0157 (6)	−0.0156 (6)
O2	0.0570 (8)	0.0457 (8)	0.0728 (10)	−0.0148 (7)	0.0342 (7)	−0.0234 (7)
N5	0.0458 (9)	0.0449 (9)	0.0513 (11)	−0.0104 (8)	0.0191 (7)	−0.0114 (8)
O3	0.0436 (8)	0.0714 (9)	0.0732 (11)	−0.0007 (7)	−0.0062 (7)	−0.0419 (8)
N3	0.0400 (9)	0.0536 (10)	0.0539 (11)	−0.0067 (8)	0.0127 (7)	−0.0193 (9)
C1	0.0410 (10)	0.0443 (10)	0.0348 (11)	−0.0065 (8)	0.0075 (8)	−0.0144 (9)
C2	0.0458 (11)	0.0436 (11)	0.0424 (11)	−0.0060 (9)	0.0030 (8)	−0.0162 (9)
C17	0.0406 (10)	0.0390 (10)	0.0426 (12)	−0.0018 (8)	0.0049 (8)	−0.0117 (9)
N4	0.0422 (9)	0.0528 (10)	0.0534 (11)	−0.0075 (8)	0.0116 (7)	−0.0200 (8)
C7	0.0509 (12)	0.0461 (11)	0.0528 (13)	0.0024 (9)	−0.0045 (9)	−0.0178 (10)
N6	0.0481 (10)	0.0530 (10)	0.0604 (12)	−0.0149 (9)	0.0221 (8)	−0.0203 (9)
C16	0.0389 (10)	0.0398 (10)	0.0396 (11)	−0.0010 (8)	0.0043 (8)	−0.0097 (9)
C18	0.0379 (10)	0.0484 (11)	0.0395 (12)	−0.0031 (9)	0.0089 (8)	−0.0110 (9)
C8	0.0406 (11)	0.0459 (11)	0.0514 (13)	−0.0130 (9)	−0.0007 (9)	−0.0147 (9)
C9	0.0472 (11)	0.0489 (11)	0.0564 (13)	−0.0068 (9)	−0.0030 (9)	−0.0175 (10)
C10	0.0468 (12)	0.0605 (13)	0.0670 (16)	−0.0124 (10)	−0.0052 (11)	−0.0140 (12)
C15	0.0588 (13)	0.0536 (12)	0.0630 (14)	−0.0063 (10)	0.0255 (10)	−0.0256 (11)
C3	0.0673 (14)	0.0433 (11)	0.0595 (14)	−0.0084 (10)	0.0009 (11)	−0.0169 (10)
C6	0.0474 (11)	0.0636 (13)	0.0448 (12)	−0.0046 (10)	0.0010 (9)	−0.0205 (10)
C13	0.0448 (11)	0.0598 (12)	0.0638 (14)	−0.0206 (10)	0.0104 (10)	−0.0271 (11)
C11	0.0621 (14)	0.0693 (14)	0.0570 (15)	−0.0264 (12)	−0.0048 (11)	−0.0167 (12)
C12	0.0643 (14)	0.0704 (14)	0.0591 (15)	−0.0340 (12)	0.0134 (11)	−0.0297 (12)
C19	0.0650 (13)	0.0486 (12)	0.0401 (12)	−0.0123 (11)	0.0133 (9)	−0.0105 (9)
C5	0.0560 (13)	0.0876 (17)	0.0600 (15)	−0.0243 (13)	0.0056 (11)	−0.0401 (13)
C4	0.0752 (15)	0.0620 (14)	0.0699 (16)	−0.0254 (13)	0.0125 (12)	−0.0346 (13)
C24	0.0824 (16)	0.0648 (15)	0.0629 (16)	−0.0224 (13)	0.0050 (12)	−0.0170 (12)
C22	0.157 (3)	0.0627 (17)	0.089 (2)	−0.027 (2)	0.025 (2)	−0.0315 (16)
C23	0.130 (3)	0.0741 (18)	0.0700 (19)	−0.0429 (18)	0.0010 (17)	−0.0207 (15)
C20	0.0751 (17)	0.0590 (15)	0.112 (2)	−0.0109 (13)	0.0269 (14)	−0.0335 (15)
C14	0.0543 (14)	0.114 (2)	0.129 (2)	−0.0165 (14)	0.0132 (14)	−0.0842 (19)
C21	0.113 (2)	0.0676 (18)	0.140 (3)	−0.0059 (18)	0.0436 (19)	−0.0384 (19)

### Geometric parameters (Å, º)

**Table:**

O1—C18	1.354 (2)	C15—H15B	0.9600
O1—C17	1.377 (2)	C15—H15C	0.9600
O2—N5	1.3912 (18)	C3—C4	1.372 (3)
O2—C15	1.422 (2)	C3—H3	0.9300
N5—C16	1.287 (2)	C6—C5	1.377 (3)
O3—C8	1.3759 (19)	C6—H6	0.9300
O3—C7	1.418 (2)	C13—C12	1.382 (3)
N3—C18	1.300 (2)	C13—C14	1.501 (3)
N3—N4	1.407 (2)	C11—C12	1.380 (3)
C1—C6	1.386 (2)	C11—H11	0.9300
C1—C2	1.397 (2)	C12—H12	0.9300
C1—C16	1.489 (2)	C19—C20	1.370 (3)
C2—C3	1.382 (2)	C19—C24	1.380 (3)
C2—C7	1.501 (2)	C5—C4	1.365 (3)
C17—N4	1.284 (2)	C5—H5	0.9300
C17—C16	1.456 (2)	C4—H4	0.9300
C7—H7A	0.9700	C24—C23	1.371 (3)
C7—H7B	0.9700	C24—H24	0.9300
N6—C18	1.339 (2)	C22—C23	1.356 (4)
N6—C19	1.406 (2)	C22—C21	1.368 (4)
N6—H6'	0.93 (2)	C22—H22	0.9300
C8—C9	1.385 (2)	C23—H23	0.9300
C8—C13	1.392 (3)	C20—C21	1.383 (3)
C9—C10	1.389 (2)	C20—H20	0.9300
C9—H9	0.9300	C14—H14A	0.9600
C10—C11	1.366 (3)	C14—H14B	0.9600
C10—H10	0.9300	C14—H14C	0.9600
C15—H15A	0.9600	C21—H21	0.9300
			
C18—O1—C17	102.10 (15)	C4—C3—C2	121.79 (18)
N5—O2—C15	109.64 (13)	C4—C3—H3	119.1
C16—N5—O2	110.37 (14)	C2—C3—H3	119.1
C8—O3—C7	117.77 (14)	C5—C6—C1	121.23 (18)
C18—N3—N4	105.98 (15)	C5—C6—H6	119.4
C6—C1—C2	119.05 (16)	C1—C6—H6	119.4
C6—C1—C16	118.34 (15)	C12—C13—C8	117.91 (18)
C2—C1—C16	122.56 (14)	C12—C13—C14	121.1 (2)
C3—C2—C1	118.43 (16)	C8—C13—C14	120.98 (17)
C3—C2—C7	119.19 (16)	C10—C11—C12	119.67 (18)
C1—C2—C7	122.38 (16)	C10—C11—H11	120.2
N4—C17—O1	112.81 (16)	C12—C11—H11	120.2
N4—C17—C16	127.54 (17)	C11—C12—C13	121.6 (2)
O1—C17—C16	119.65 (16)	C11—C12—H12	119.2
C17—N4—N3	106.21 (14)	C13—C12—H12	119.2
O3—C7—C2	108.75 (15)	C20—C19—C24	119.4 (2)
O3—C7—H7A	109.9	C20—C19—N6	123.6 (2)
C2—C7—H7A	109.9	C24—C19—N6	116.96 (19)
O3—C7—H7B	109.9	C4—C5—C6	119.65 (17)
C2—C7—H7B	109.9	C4—C5—H5	120.2
H7A—C7—H7B	108.3	C6—C5—H5	120.2
C18—N6—C19	129.13 (18)	C5—C4—C3	119.83 (19)
C18—N6—H6'	111.4 (14)	C5—C4—H4	120.1
C19—N6—H6'	118.8 (14)	C3—C4—H4	120.1
N5—C16—C17	115.10 (16)	C23—C24—C19	120.3 (2)
N5—C16—C1	126.20 (17)	C23—C24—H24	119.9
C17—C16—C1	118.63 (16)	C19—C24—H24	119.9
N3—C18—N6	125.74 (18)	C23—C22—C21	119.2 (3)
N3—C18—O1	112.89 (17)	C23—C22—H22	120.4
N6—C18—O1	121.25 (18)	C21—C22—H22	120.4
O3—C8—C9	123.67 (17)	C22—C23—C24	120.8 (3)
O3—C8—C13	115.14 (16)	C22—C23—H23	119.6
C9—C8—C13	121.19 (17)	C24—C23—H23	119.6
C8—C9—C10	119.03 (19)	C19—C20—C21	119.3 (3)
C8—C9—H9	120.5	C19—C20—H20	120.3
C10—C9—H9	120.5	C21—C20—H20	120.3
C11—C10—C9	120.60 (19)	C13—C14—H14A	109.5
C11—C10—H10	119.7	C13—C14—H14B	109.5
C9—C10—H10	119.7	H14A—C14—H14B	109.5
O2—C15—H15A	109.5	C13—C14—H14C	109.5
O2—C15—H15B	109.5	H14A—C14—H14C	109.5
H15A—C15—H15B	109.5	H14B—C14—H14C	109.5
O2—C15—H15C	109.5	C22—C21—C20	121.1 (3)
H15A—C15—H15C	109.5	C22—C21—H21	119.5
H15B—C15—H15C	109.5	C20—C21—H21	119.5
			
C15—O2—N5—C16	176.64 (15)	C7—O3—C8—C13	−178.30 (18)
C6—C1—C2—C3	0.1 (3)	O3—C8—C9—C10	−178.63 (18)
C16—C1—C2—C3	177.78 (18)	C13—C8—C9—C10	0.6 (3)
C6—C1—C2—C7	179.86 (17)	C8—C9—C10—C11	1.0 (3)
C16—C1—C2—C7	−2.4 (3)	C1—C2—C3—C4	0.8 (3)
C18—O1—C17—N4	−0.61 (19)	C7—C2—C3—C4	−178.94 (19)
C18—O1—C17—C16	179.83 (15)	C2—C1—C6—C5	−1.3 (3)
O1—C17—N4—N3	−0.39 (19)	C16—C1—C6—C5	−179.06 (18)
C16—C17—N4—N3	179.14 (16)	O3—C8—C13—C12	177.67 (17)
C18—N3—N4—C17	1.28 (19)	C9—C8—C13—C12	−1.7 (3)
C8—O3—C7—C2	174.85 (15)	O3—C8—C13—C14	−2.7 (3)
C3—C2—C7—O3	−120.2 (2)	C9—C8—C13—C14	177.93 (19)
C1—C2—C7—O3	60.0 (2)	C9—C10—C11—C12	−1.6 (3)
O2—N5—C16—C17	179.34 (14)	C10—C11—C12—C13	0.5 (3)
O2—N5—C16—C1	2.2 (2)	C8—C13—C12—C11	1.1 (3)
N4—C17—C16—N5	−164.43 (18)	C14—C13—C12—C11	−178.5 (2)
O1—C17—C16—N5	15.1 (2)	C18—N6—C19—C20	−14.8 (3)
N4—C17—C16—C1	12.9 (3)	C18—N6—C19—C24	167.7 (2)
O1—C17—C16—C1	−167.60 (14)	C1—C6—C5—C4	1.5 (3)
C6—C1—C16—N5	63.0 (3)	C6—C5—C4—C3	−0.6 (3)
C2—C1—C16—N5	−114.7 (2)	C2—C3—C4—C5	−0.6 (3)
C6—C1—C16—C17	−114.00 (19)	C20—C19—C24—C23	0.2 (3)
C2—C1—C16—C17	68.3 (2)	N6—C19—C24—C23	177.8 (2)
N4—N3—C18—N6	174.36 (17)	C21—C22—C23—C24	−0.4 (4)
N4—N3—C18—O1	−1.76 (19)	C19—C24—C23—C22	0.5 (4)
C19—N6—C18—N3	162.55 (19)	C24—C19—C20—C21	−1.0 (4)
C19—N6—C18—O1	−21.6 (3)	N6—C19—C20—C21	−178.5 (2)
C17—O1—C18—N3	1.49 (19)	C23—C22—C21—C20	−0.5 (5)
C17—O1—C18—N6	−174.82 (16)	C19—C20—C21—C22	1.2 (4)
C7—O3—C8—C9	1.0 (3)		

### Hydrogen-bond geometry (Å, º)

**Table:**

*D*—H···*A*	*D*—H	H···*A*	*D*···*A*	*D*—H···*A*
N6—H6′···N3^i^	0.93 (2)	1.99 (2)	2.922 (2)	174

Symmetry code: (i) −*x*+2, −*y*+1, −*z*.
